# Complete intra-laboratory validation of a LAL assay for bacterial endotoxin determination in EBV-specific cytotoxic T lymphocytes

**DOI:** 10.1016/j.omtm.2021.05.002

**Published:** 2021-05-14

**Authors:** Salvatore Pasqua, Maria Concetta Niotta, Giuseppina Di Martino, Davide Sottile, Bruno Douradinha, Monica Miele, Francesca Timoneri, Mariangela Di Bella, Nicola Cuscino, Chiara Di Bartolo, Pier Giulio Conaldi, Danilo D’Apolito

**Affiliations:** 1 Unità Prodotti Cellulari (GMP), Fondazione Ri.MED c/o IRCCS-ISMETT (Istituto Mediterraneo per i Trapianti e Terapie ad Alta Specializzazione), Via E. Tricomi 5, 90127 Palermo, Italy; 2 Unità di Medicina di Laboratorio e Biotecnologie Avanzate, IRCCS-ISMETT (Istituto Mediterraneo per i Trapianti e Terapie ad Alta Specializzazione), Via E. Tricomi 5, 90127 Palermo, Italy; 3 Unità Medicina Rigenerativa ed Immunologia, Fondazione Ri.MED c/o IRCCS-ISMETT (Istituto Mediterraneo per i Trapianti e Terapie ad Alta Specializzazione), Via E. Tricomi 5, 90127 Palermo, Italy; 4 Ufficio Ricerca, IRCCS-ISMETT (Istituto Mediterraneo per i Trapianti e Terapie ad Alta Specializzazione), Via E. Tricomi 5, 90127 Palermo, Italy

**Keywords:** advanced therapy medicinal products, ATMPs, endotoxins, qualification, method validation, *Limulus* amebocyte lysate, LAL, failure mode and effects analysis, FMEA, GMP, good manufacturing practices

## Abstract

Endotoxin content is a critical factor that affects the safety of biological pharmaceutical products. International pharmacopoeias describe several reference methods to determine endotoxin levels in advanced therapy medicinal product (ATMP) preparations. Administration of ATMPs must be done as rapidly as possible to ensure complete viability and potency of the cellular product. To evaluate the endotoxin content in the shortest time possible, we chose to validate an alternative method based on the use of the Charles River Portable Testing System (PTS) and FDA-approved cartridges, compliant with the requirements of the European Pharmacopoeia and providing results in <20 min. Here, we describe a unique and complete validation approach for instrument, personnel, and analytical method for assessment of endotoxins in ATMP matrices. The PTS system provides high sensitivity and fast quantitative results and uses less raw material and accessories compared with compendial methods. It is also less time consuming and less prone to operator variability. Our validation approach is suitable for a validated laboratory with trained personnel capable of conducting the ATMP release tests, and with very low intra-laboratory variability, and meets the criteria required for an alternative approach to endotoxin detection for in-process and product-release testing of ATMPs.

## Graphical abstract

**Figure:**
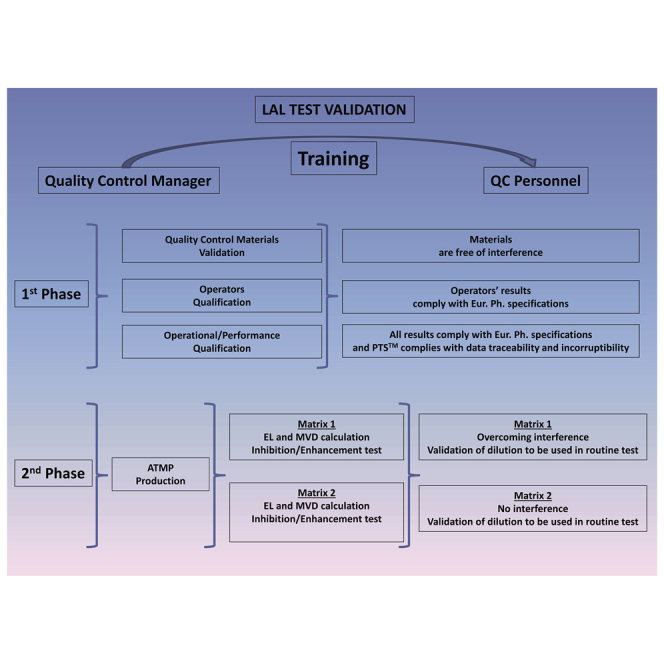

## Teaser

Endotoxin assessment is crucial to ensure that ATMP administration to patients can be done as soon and as safely as possible. The use of PTS allows us to obtain fast, robust, and reproducible results, and its application simplifies the related procedures in a time- and money-saving manner.

## Introduction

Advanced therapy medicinal products (ATMPs) must be prepared according to good manufacturing practices (GMP).^1^, ^2^, ^3^, ^4^ The release strategy of an ATMP involves the execution of analytical methods that evaluate safety, identity, purity, and potency of the final product to be administered to the patient. Among them, evaluation of endotoxin presence is crucial, as required by section 2.6.14 of the European Pharmacopoeia (Eur. Ph.).^5^ Endotoxins from gram-negative bacteria are the most common cause of toxic reactions, resulting in the contamination of pharmaceutical products with pyrogens. Their pyrogenic activity is much higher than that of other pyrogenic substances.^6^ The pathological effects of endotoxins are a rapid increase in core body temperature followed by extremely rapid and severe shock, often mortal before it is even diagnosed.^7^ Except in cases of bacterial contamination, the main sources of endotoxins are the materials and media used during the production and control phases, which obviously have contact with the product. For these reasons, it is important that they have a very low endotoxinic content, minimizing the overall endotoxin levels present during the analytical release test and in the final product. The Eur. Ph. describes six methods to evaluate the presence of endotoxins in biological samples.^5^ To develop an endotoxin evaluation test based on the Eur. Ph. kinetic chromogenic method, we chose the Endosafe Portable Testing System (PTS) reader, which is a rapid manual spectrophotometer that uses cartridges for accurate, convenient, and real-time endotoxin testing. The Endosafe cartridge is an innovative technology that provides higher sensitivity and faster quantitative results. Designed to optimize and refine the use of the lyophilized amebocyte lysate (LAL) test to measure gram-negative bacteria endotoxins, the cartridges eliminate the need for significant quantities of raw materials and accessories required for traditional LAL methods while reducing time-consuming preparation and operator variability.^8^ Traditional endotoxin tests have pain points such as long turnaround times, extensive operator training, and multiple steps for assay preparation, increasing the possibility of obtaining false positive results that, in the worst case, can potentially cripple manufacturing timelines.^8^ Here, we describe a qualified and validated strategy for assessment of bacterial endotoxin levels in ATMPs, which complies with GMP guidelines^1^ and Eur. Ph.^5^

## Results

### Installation qualification protocol

Upon arrival of the PTS reader (Charles River, Wilmington, MA, USA), which was previously submitted to a qualification protocol (QP) by the supplier, we executed the installation qualification (IQ) protocol. All documentation and certifications of the instrument were present, such as calibration certificates of the PTS reader, certificates of analysis of LAL reagent water and cartridges used during QP, and results obtained by the supplier during QP and before its delivery. Subsequently, we have reported in our internal forms all the information concerning the instrument and respective supplied accessories, including the serial numbers and the reference codes. After completing these initial control steps and confirming that they complied with the expected specifications, we connected the instrument to the power outlet, turned it on, and recorded the information of the installed software in our internal forms. Finally, we connected the printer to the PTS reader and verified that the connection was functional.

### Training, validation of materials, qualification of operators, and operational/performance qualification

After having trained the personnel, we performed the operative protocol that simultaneously executes the validation of the materials, the qualification of the operators, and the operational/performance qualification (OPQ) of the instrument. Initially, each operator validated a different lot of cartridges, performing the test on 3 sample cartridges, using the materials we selected, and loading 25 μL of LAL water in each well (Table 1).

**Table 1:** Validation of materials and qualification of operators

Operator	Sample	Spike rxn time CV^a^	Spike recovery	Expected value	Measured value	Average	Standard deviation
QC manager	LAL water 1	0.5% pass	71% pass	<0.005 EU/mL	<0.005 EU/mL	<0.005 EU/mL	0
	LAL water 2	1.1% pass	74% pass	<0.005 EU/mL	<0.005 EU/mL		
	LAL water 3	3.3% pass	82% pass	<0.005 EU/mL	<0.005 EU/mL		
Operator #1	LAL water 1	2.9% pass	96% pass	<0.005 EU/mL	<0.005 EU/mL	<0.005 EU/mL	0
	LAL water 2	4.0% pass	66% pass	<0.005 EU/mL	<0.005 EU/mL		
	LAL water 3	1.5% pass	103% pass	<0.005 EU/mL	<0.005 EU/mL		
Operator #2	LAL water 1	3.6% pass	75% pass	<0.005 EU/mL	<0.005 EU/mL	<0.005 EU/mL	0
	LAL water 2	3.9% pass	74% pass	<0.005 EU/mL	<0.005 EU/mL		
	LAL water 3	3.9% pass	62% pass	<0.005 EU/mL	<0.005 EU/mL		
Operator #3	LAL water 1	6.5% pass	78% pass	<0.005 EU/mL	<0.005 EU/mL	<0.005 EU/mL	0
	LAL water 2	6.1% pass	84% pass	<0.005 EU/mL	<0.005 EU/mL		
	LAL water 3	3.3% pass	67% pass	<0.005 EU/mL	<0.005 EU/mL		

a Spike rxn time CV, spike reaction time coefficient of variation.

In each experiment performed by an individual operator, all the information submitted to the instrument before loading the samples was displayed in the final receipt, which contained the experiment results, confirming that the PTS worked well and kept all data entered in the pre-analytical phase. We observed that the results of the experiments were compliant with what was expected and the pH value was always between 6 and 8, specifically between 6.9 and 7.1. Our results also confirm the ability of the operators to correctly load the samples inside the cartridge reservoirs and to obtain valid and repeatable results. Also, no interfering activity derived from the materials was observed. The qualification of operators and OPQ results obtained with reference standard endotoxin (RSE) dilution suspensions (Table 2) are summarized in Table 3. The results obtained also showed that the operators can correctly prepare the samples and serial dilution suspensions of the RSE and load them into the cartridges, obtaining robust and repeatable recovery results within the range provided by the method (Table 3).

**Table 2:** Serial dilutions of reference standard endotoxin (RSE)

Dilution	Endotoxin concentration (EU/mL)	Volume of LAL water	Volume of endotoxin added to LAL water
A	2,000	5 mL	Powder
B	200	1.8 mL	0.2 mL of dilution A
C	20	1.8 mL	0.2 mL of dilution B
D	2	1.8 mL	0.2 mL of dilution C
E	0.2	1.8 mL	0.2 mL of dilution D
F	0.02	1.8 mL	0.2 mL of dilution E
G	0.05	1.5 mL	0.5 mL of dilution E
H	0.01	1.0 mL	1.0 mL of dilution F

**Table 3:** Operational/performance qualification results

Operator	RSE dilution suspension	Spike rxn time CV	Spike recovery	Expected value	Measured value	Average	Standard deviation	RSE recovery %
QC manager	F	1.2% pass	83% pass	<0.02 EU/mL	0.016 EU/mL	0.015	8.16 × 10^−4^	80
		1.2% pass	88% pass	<0.02 EU/mL	0.014 EU/mL			70
		2.4% pass	59% pass	<0.02 EU/mL	0.015 EU/mL			75
	G	1.3% pass	84% pass	<0.05 EU/mL	0.039 EU/mL	0.033	4.32 × 10^−3^	78
		4.7% pass	97% pass	<0.05 EU/mL	0.029 EU/mL			58
		1.8% pass	83% pass	<0.05 EU/mL	0.031 EU/mL			62
	H	6.3% pass	97% pass	<0.01 EU/mL	0.013 EU/mL	0.013	8.16 × 10^−4^	130
		0.3% pass	81% pass	<0.01 EU/mL	0.014 EU/mL			140
		4.1% pass	93% pass	<0.01 EU/mL	0.012 EU/mL			120
Operator #1	F	10.5% pass	114% pass	<0.02 EU/mL	0.018 EU/mL	0.018	4.71 × 10^−4^	90
		0.6% pass	121% pass	<0.02 EU/mL	0.019 EU/mL			95
		3.2% pass	116% pass	<0.02 EU/mL	0.018 EU/mL			90
	G	4.9% pass	90% pass	<0.05 EU/mL	0.009 EU/mL	0.009	0.00	90
		14.4% pass	142% pass	<0.05 EU/mL	0.009 EU/mL			90
		9.5% pass	72% pass	<0.05 EU/mL	0.009 EU/mL			90
	H	11.0% pass	100% pass	<0.01 EU/mL	0.013 EU/mL	0.013	8.16 × 10^−4^	130
		0.9% pass	98% pass	<0.01 EU/mL	0.014 EU/mL			140
		6.4% pass	88% pass	<0.01 EU/mL	0.012 EU/mL			120
Operator #2	F	2.2% pass	104% pass	<0.02 EU/mL	0.017 EU/mL	0.017	4.71 × 10^−4^	85
		0.3% pass	92% pass	<0.02 EU/mL	0.017 EU/mL			85
		0.0% pass	93% pass	<0.02 EU/mL	0.016 EU/mL			80
	G	0.3% pass	92% pass	<0.05 EU/mL	0.055 EU/mL	0.049	5.31 × 10^−3^	110
		3.3% pass	107% pass	<0.05 EU/mL	0.042 EU/mL			84
		1.6% pass	133% pass	<0.05 EU/mL	0.049 EU/mL			98
	H	4.5% pass	83% pass	<0.01 EU/mL	0.008 EU/mL	0.010	1.25 × 10^−3^	80
		1.6% pass	105% pass	<0.01 EU/mL	0.010 EU/mL			100
		0.6% pass	158% pass	<0.01 EU/mL	0.011 EU/mL			110
Operator #3	F	8.1% pass	71% pass	<0.02 EU/mL	0.015 EU/mL	0.021	4.92 × 10^−3^	75
		7.4% pass	78% pass	<0.02 EU/mL	0.027 EU/mL			135
		2.6% pass	113% pass	<0.02 EU/mL	0.020 EU/mL			100
	G	7.0% pass	82% pass	<0.05 EU/mL	0.046 EU/mL	0.042	3.30 × 10^−3^	92
		11.2% pass	106% pass	<0.05 EU/mL	0.043 EU/mL			86
		7.8% pass	105% pass	<0.05 EU/mL	0.038 EU/mL			76
	H	4.9% pass	60% pass	<0.01 EU/mL	0.007 EU/mL	0.010	2.45 × 10^−3^	70
		3.8% pass	95% pass	<0.01 EU/mL	0.013 EU/mL			130
		13.2% pass	72% pass	<0.01 EU/mL	0.010 EU/mL			100

### Estimation of endotoxin limit (EL) and maximum valid dilution (MVD) of the matrices

After the qualification of the instrument and operators and the validation of materials, we proceeded with the validation of the analytical method, evaluating the possible interference of the matrices described in Table 4. For estimating the EL and the MVD, we considered a value of 20 kg as a reference weight, which represents the average weight of children with post-transplant lymphoproliferative disease (PTLD). As detailed below in Materials and methods, EL is defined as the number of endotoxin units (EU) allowed per milliliter of product, or

EL= K/M

where K is a constant and as specified by Ph. Eur. is 5 EU/kg^5^^,^^9^ and M is the maximum cellular dose infused per kilogram in a single hour period and in particular the maximum volume of infusion per kilogram.^5^^,^^9^ For Matrix 1, considering 40 mL the maximum volume of infusion equal to the cell washing medium and 20 kg the reference weight,

M=40 mL÷20 kg=2 mL/kg

Thus, as observed in Table 4, EL of Matrix 1 is

EL=5 EU/kg ÷2 mL/kg=2.5 EU/mL .

The MVD is the product maximum dilution at which the EL can be determined:

$$\text{MVD}=\frac{\text{EL }\times \text{ C}}{\text{λ}}$$

It is defined by the product of EL and the concentration C divided by the lowest point of the cartridge standard curve, or λ. C refers to the concentration of the solution to be tested, and as described in Materials and methods, is equal to 1. The lowest point of the standard curve for the cartridge used in our assays was 0.005 EU/mL.

**Table 4:** Endotoxin limit (EL) and maximum valid dilution (MVD) of matrices

Matrix	EL	MVD
Pre-infusion supernatant (frozen cell washing supernatant; Matrix 1)	2.5 EU/mL	500
Pre-freezing supernatant (culture medium; Matrix 2)	2.22 EU/mL	444

Thus, for Matrix 1, as observed in Table 4, the MVD is

$$\text{MVD}=\frac{2.5\text{ EU}/\text{mL }\times 1}{0.005\text{ EU}/\text{mL }}=500$$

Similarly, for Matrix 2, considering a volume of 45 mL, EL and MVD are 2.22 EU/mL and 444, respectively (Table 4).

### Validation of the analytical method for both matrices

As required by the validation protocol, we tested 3 batches of the matrices at the planned dilutions. For each batch, we used a different lot of previously validated cartridges. For Matrix 1, the results obtained did not respect the expected acceptability criteria even at MVD/2 and MVD, because of interfering activity of the albumin present in the buffer (data not shown). Therefore, samples were diluted in a specific reagent buffer, BG120, to eliminate these interferences. This buffer contains a high concentration of carboxymethylated curdlan, and Charles River suggests its use to prevent the activation of the Factor G zymogen in LAL.^10^^,^^11^ We then tested the 3 batches of Matrix 1 at the expected dilutions, performing a prior 1:2 dilution with the BG120 buffer. Treatment with BG120 eliminated the interferences, since acceptability criteria in all 3 lots of Matrix 1 were observed in all tests performed at MVD/2 and MVD, showing reproducible and robust data and the validity of the tests (Table 5). Consequently we continued the validation of the method to identify the 3 non-interfering dilutions within the undiluted–MVD/2 dilution range. As before, the BG120 buffer was used. The results of the analysis performed in duplicate are described in Table 6. We observed that 1:125 is the third non-interfering dilution, which must be used to do the routine analyses of Matrix 1. For this dilution, all results showed endotoxin values lower than 0.625 EU/ml, which is well below the expected value of 2.5 EU/ml, demonstrating that the materials and reagents used in the preparation of this matrix barely contributed to the total level of endotoxins.

**Table 5:** Matrix 1 validation results

Operator	Matrix	Sample	Spike rxn time CV	Spike recovery	Measured value	Average	Standard deviation
QC manager	M1 Lot A	undiluted	2.4% pass	2% fail	<0.005 EU/mL	<0.005 EU/mL	0
			2.2% pass	2% fail	<0.005 EU/mL		
		MVD/2 (1:250)	0.5% pass	65% pass	<1.25 EU/mL	<1.25 EU/mL	0
			2.7% pass	65% pass	<1.25 EU/mL		
		MVD (1:500)	4.7% pass	96% pass	<2.5 EU/mL	<2.5 EU/mL	0
			3.0% pass	86% pass	<2.5 EU/mL		
Operator #1	M1 Lot B	undiluted	0.2% pass	4% fail	< 0.005 EU/mL	<0.005 EU/mL	0
			2.5% pass	5% fail	<0.005 EU/mL		
		MVD/2 (1:250)	3.6% pass	61% pass	<1.25 EU/mL	<1.25 EU/mL	0
			0.4% pass	63% pass	<1.25 EU/mL		
		MVD (1:500)	0.0% pass	76% pass	<2.5 EU/mL	<2.5 EU/mL	0
			2.9% pass	77% pass	<2.5 EU/mL		
Operator #2	M1 Lot C	undiluted	20.5% pass	10% fail	<0.007 EU/mL	<0.0065 EU/mL	0.0005
			1.5% pass	3% fail	<0.006 EU/mL		
		MVD/2 (1:250)	2.0% pass	81% pass	<1.25 EU/mL	<1.25 EU/mL	0
			1.0% pass	82% pass	<1.25 EU/mL		
		MVD (1:500)	2.8% pass	74% pass	<2.5 EU/mL	<2.5 EU/mL	0
			3.2% pass	69% pass	<2.5 EU/mL

**Table 6:** Matrix 1 validation results and determination of the 3 non-interfering dilutions within the MVD/2 dilution

Operator	Matrix	Sample	Spike rxn time CV	Spike recovery	Measured value	Average	Standard deviation
QC manager	M1 Lot A	1:75	9.6% pass	77% pass	<0.375 EU/mL	<0.375 EU/mL	0
			4.3% pass	54% pass	<0.375 EU/mL		
		1:100	3.3% pass	72% pass	<0.5 EU/mL	<0.5 EU/mL	0
			0.0% pass	61% pass	<0.5 EU/mL		
		1:125	0.0% pass	70% pass	<0.625 EU/mL	<0.625 EU/mL	0
			1.7% pass	56% pass	<0.625 EU/mL		
Operator #1	M1 Lot B	1:75	5.1% pass	68% pass	<0.375 EU/mL	<0.375 EU/mL	0
			6.9% pass	68% pass	<0.375 EU/mL		
		1:100	1.3% pass	52% pass	<0.5 EU/mL	<0.5 EU/mL	0
			12.3% pass	74% pass	<0.5 EU/mL		
		1:125	9.6% pass	67% pass	<0.625 EU/mL	<0.625 EU/mL	0
			5.1% pass	69% pass	<0.625 EU/mL		
Operator #2	M1 Lot C	1:75	1.4% pass	68% pass	<0.375 EU/mL	<0.375 EU/mL	0
			4.8% pass	57% pass	<0.375 EU/mL		
		1:100	2.8% pass	70% pass	<0.5 EU/mL	<0.5 EU/mL	0
			4.8% pass	59% pass	< 0.5 EU/mL		
		1:125	2.7% pass	61% pass	<0.625 EU/mL	<0.625 EU/mL	0
			2.5% pass	52% pass	<0.625 EU/mL		

Validation of Matrix 2 was performed similarly, testing 3 different batches at the pre-defined dilutions and with different lots of validated cartridges. To simplify the dilution procedure, we chose an MVD limit of 400 instead of the previously determined value of 444 (Table 4). The results obtained by analyzing Matrix 2 respect the expected acceptability criteria (Table 7), confirming the validity of the test and that the matrix did not have interfering activity. Unsurprisingly, the test suggested the presence of interference factors in the undiluted sample, leading to no spike recovery (0% fail) and, consequently, to the failure of the test (Table 7). Since the results obtained using the MVD/2 and MVD dilutions were valid, we continued the validation of the method to identify the 3 non-interfering dilutions within the undiluted–MVD/2 dilution range (Table 8). We observed that the dilution 1:40 is the third non-interfering dilution, i.e., the one to be considered while doing the analyses of Matrix 2. For this dilution, endotoxin values were lower than 0.108 EU/ml, which is lower than the expected value of 2.22 EU/ml for this matrix. Thus, as stated before for Matrix 1, materials and reagents also scarcely contributed to the overall levels of endotoxins.

**Table 7:** Matrix 2 validation

Operator	Matrix	Sample	Spike rxn time CV	Spike recovery	Measured value	Average	Standard deviation
QC manager	M2 Lot A	undiluted	0.0% pass	0% fail	<0.005 EU/mL	<0.005 EU/mL	0
			0.0% pass	0% fail	<0.005 EU/mL		
		MVD/2 (1:200)	0.0% pass	57% pass	<1 EU/mL	<1 EU/mL	0
			1.3% pass	82% pass	<1 EU/mL		
		MVD (1:400)	2.8% pass	72% pass	<2 EU/mL	<2 EU/mL	0
			1% pass	112% pass	<2 EU/mL		
Operator #1	M2 Lot B	undiluted	0.0% pass	0% fail	<0.005 EU/mL	<0.005 EU/mL	0
			0.0% pass	0% fail	<0.005 EU/mL		
		MVD/2 (1:200)	0.7% pass	98% pass	<1 EU/mL	<1 EU/mL	0
			3.8% pass	64% pass	<1 EU/mL		
		MVD (1:400)	3.7% pass	73% pass	<2 EU/mL	<2 EU/mL	0
			1.7% pass	116% pass	<2 EU/mL		
Operator #2	M2 Lot C	undiluted	0.0% pass	0% fail	<0.005 EU/mL	<0.005 EU/mL	0
			0.0% pass	0% fail	<0.005 EU/mL		
		MVD/2 (1:200)	2.5% pass	64% pass	<1 EU/mL	<1 EU/mL	0
			0.2% pass	81% pass	<1 EU/mL		
		MVD (1:400)	0.2% pass	79% pass	<2 EU/mL	<2 EU/mL	0
			2.7% pass	91% pass	<2 EU/mL

**Table 8:** Matrix 2 validation and determination of the 3 non-interfering dilutions within the MVD/2 dilution

Operator	Matrix	Sample	Spike rxn time CV	Spike recovery	Measured value	Average	Standard deviation
QC manager	M2 Lot A	1:10	0.7% pass	87% pass	<0.05 EU/mL	<0.05 EU/mL	0
			3.4% pass	71% pass	<0.05 EU/mL		
		1:20	2.5% pass	57% pass	<0.1 EU/mL	<0.1 EU/mL	0
			11% pass	114% pass	<0.1 EU/mL		
		1:40	3.8% pass	83% pass	<0.2 EU/mL	<0.2 EU/mL	0
			1.1% pass	75% pass	<0.2 EU/mL		
Operator #1	M2 Lot B	1:10	3.0% pass	73% pass	<0.05 EU/mL	<0.05 EU/mL	0
			6.1% pass	55% pass	<0.05 EU/mL		
		1:20	1.7% pass	75% pass	<0.1 EU/mL	<0.1 EU/mL	0
			0.6% pass	71% pass	<0.1 EU/mL		
		1:40	3.2% pass	69% pass	<0.2 EU/mL	<0.2 EU/mL	0
			1.9% pass	67% pass	<0.2 EU/mL		
Operator #2	M2 Lot C	1:10	0.0% pass	60% pass	<0.05 EU/mL	<0.05 EU/mL	0
			1.8% pass	84% pass	<0.05 EU/mL		
		1:20	3.0% pass	73% pass	<0.1 EU/mL	<0.108 EU/mL	0.008
			3.1% pass	118% pass	<0.116 EU/mL		
		1:40	13.1% pass	137% pass	<0.2 EU/mL	<0.2 EU/mL	0
			1.7% pass	62% pass	<0.2 EU/mL		

## Discussion

Before releasing an ATMP, several tests must be performed, including evaluation of endotoxin levels. The Eur. Ph. recommends 6 different analytical methods for endotoxin detection,^5^ which are time-consuming and labor-intensive. To overcome these critical aspects, Charles River developed an automatic system to detect the presence of endotoxins, the Endosafe PTS reader. This system and the respective cartridges were designed to perform the currently licensed endpoint chromogenic and kinetic chromogenic methods by measuring color intensity directly related to the endotoxin concentration in a sample.^8^ Each cartridge contains precise amounts of Food and Drug Administration (FDA)-licensed LAL formulations, a chromogenic substrate, and control standard endotoxin (CSE).^8^ The PTS reader is a tool that possesses exhaustive documentation, a user-friendly approach procedure, and high-quality accessories and materials.

Our results obtained using this technology gave reproducible and robust results in <20 min. This is desirable, since time is a crucial aspect for ATMPs, especially those that require a rapid release for immediate administration into the patient. The Endosafe PTS reader was previously evaluated for its use in endotoxin detection, showing robust and reproducible results.^9^^,^^12^, ^13^, ^14^ Because of its promising innovative and technological features, we chose the PTS reader to evaluate the presence of endotoxin in ATMP in-house products.

The initial execution of the qualifications required by GMP regulations and, in particular the IQ and the OPQ, confirmed that the supplier provides a fully qualified and ready-to-use instrument and that the respective documentation is complete and exhaustive, as demanded by regulatory and inspection agencies. Moreover, the results of the performance qualification, done in parallel with the qualification of the operators and the validation of the chosen materials, showed that the PTS reader did not lose or change any of the information inserted in the instrument during the pre-analytical phase, complying with the fundamental GMP guidelines for data traceability and incorruptibility.^15^^,^^16^ Also, the tests performed confirmed that the materials were compliant with the methodology used and that an adequate and short training, due to few procedural steps, allowed the operators to obtain highly reproducible and robust results in a very rapid way. Subsequently, we validated the analytical method, as required by the Eur. Ph.^5^ A fundamental aspect in validation of assays to determine LAL levels in pharmaceutical drugs is the determination of EL and MVD. However, international guidelines do not provide a clear definition of how to assess these parameters in ATMPs. This has led to a non-uniformity of calculation of these parameters.^7^^,^^13^ Some authors have used EL and MVD values without clarifying how they were calculated,^13^ whereas others, having considered their ATMPs either general medical devices or individual pharmaceutical products that have a maximum human dose of 10 mL/kg,^7^ have chosen the EL value of 0.5 EU/mL suggested by FDA.^17^ In our work, we calculated the EL and MVD of our products based on their future clinical use. Their estimation, for both matrices, showed that they possessed different characteristics. Matrix 2, consisting of the cell culture supernatant, did not show interfering activity, most probably because of the absence of albumin and other interfering substances. On the other hand, Matrix 1 had to be pre-treated with BG120 to eliminate the albumin’s interference, as required by the Eur. Ph. to ensure that potential interferences present in the sample are reduced or eliminated.^5^

Both matrices displayed endotoxin levels below their expected value, for all batches tested and for all operators (Tables 5, 6, 7, and 8). All the analyses confirmed the robustness and reproducibility of the method and showed values well below the calculated theoretical values, demonstrating how the choice of certified GMP grade materials does not contribute greatly to the overall final endotoxin values in ATMPs, thus ensuring patient safety. Moreover, as suggested by regulatory agencies, we performed a risk analysis using failure mode and effects analysis (FMEA),^18^, ^19^, ^20^ further confirming that the PTS is adequate for quality control operations, because of its low possibility of showing failure modes and, if these are detected, the ease with which they are corrected (Table S1).

The robustness was also confirmed by the trend analysis of the results obtained over 3 years on several production batches, which demonstrated that our validated method allows us to obtain fast and robust results.

### Conclusions

In conclusion, we demonstrated the development and validation of a LAL test for a GMP quality control laboratory using the PTS reader and its cartridges. The test is suitable to quantify the endotoxin levels in ATMPs, and its application simplifies the related procedures in a time- and money-saving manner. The increasing interest of international regulations in using the LAL assay to reduce and replace animal testing^21^ and our successful LAL method validation confirmed that this instrument is a useful tool to evaluate the safety of ATMPs.

We believe this approach can be used by researchers involved in quality control activities, who need to set up and validate an easy and fast method for endotoxin detection that guarantees patient safety.

## Materials and methods

### The Endosafe portable testing system

The PTS reader uses cartridges to perform the kinetic-chromogenic method mentioned by the Eur. Ph.,^5^ which is based on the development of color by the sample-lysate mixture and is directly related to the endotoxin concentration in a sample. The reaction takes place at the temperature recommended by the lysate manufacturer, usually within a range of 37°C ± 1°C, and, in particular, the time necessary to reach a certain level of absorbance is measured. Cartridges are manufactured according to rigid standard operating procedures, such as test accuracy, consistency, and product stability. The cartridges are licensed by FDA and accepted by United States Pharmacopoeia (USP) and Eur. Ph. for testing raw materials, in-process samples, and final products.^5^^,^^6^^,^^22^ The cartridges contain precise amounts of LAL reagents, chromogenic substrate, and CSE loaded into wells. The cartridges contain 2 sample wells and 2 spiked wells, which allows the tests to be done automatically in duplicate, thus satisfying the harmonized USP/Eur. Ph. bacterial endotoxin test (BET) for LAL assessment. To perform a test, the user simply pipettes 25 μL of a sample into each of the four sample reservoirs of the cartridge. The reader mixes the sample with the LAL reagent, in addition to the positive control in the spike channels. The chromogenic substrate is then automatically added and incubated together with the sample-LAL reagent mixture. After mixing, the optical density of the wells is measured and analyzed against an internally archived standard curve, to determine the endotoxin value. This curve is prepared and determined by the manufacturer for each lot of cartridges, and all information is stored within its calibration code, which is reported on their certificate of analysis associated and uploaded into the instrument on the first use. The curve is generated using 5 cartridges for each sensitivity range (10–0.1, 5–0.05, 1–0.01 EU/mL), which means 10 replicates for each range, which exceeds the minimum requirements demanded by Eur. Ph. Also, the manufacturer’s criterion of acceptability of the correlation coefficient is more restrictive than the Eur. Ph. itself, stating that the PTS reader standard curve must have a correlation coefficient ≥ 0.990. Results are displayed on the LCD screen and printed. Eur. Ph. acceptance criterion for a valid assay is a curve correlation coefficient higher than 0.980, a coefficient of variation of the reaction time for the replicas smaller than 25%, and a positive product control (PPC) spike recovery of 50%–200%.^8^

### Material selection

The guidelines for the LAL test^5^ require the use of products that are free of detectable endotoxins and free of interference, e.g., from released plastic.^8^ We chose disposable, certified materials that had the lowest possible endotoxinic content and were single-packed, to minimize both contaminations and interferences and to be able to use them in a cleanroom area. The quality control materials used in this work are listed in Table 9.

**Table 9:** Quality control materials used in this work

Material	Supplier	Endotoxin content (EU/mL)
Polypropylene safe lock tube	Eppendorf	<0.001
Tips	Eppendorf	<0.001
LAL water	Charles River	<0.001
Tips	Charles River	<0.005
BG120	Charles River	<0.005

### Qualification protocol and installation qualification

Prior to the delivery of the PTS reader, Charles River performs a QP to ensure that the instrument works according to GMP guidelines.^1^^,^^23^ Our IQ protocol was to verify that all certified documents and equipment were present and that the PTS switched on after connection to the power outlet. All these verification and control phases were reported in our internal forms.

### Training, validation of materials, qualification of operators, and operational/performance qualification

We designed and developed an experimental protocol to qualify simultaneously the PTS reader, the materials used, and the operators’ training. The use of the PTS reader allowed rapid training of the personnel, highlighting the importance of correctly mixing the samples by vortexing them and by adequate loading of the sample into the wells of the cartridge, of using calibrated micropipettes and by placing the tips in an angle of ∼30–45°.

A validation protocol approach to qualify operators and the PTS reader was designed, and the operators were trained.

Initially, each operator validated a batch of cartridges, testing 3 cartridges with LAL water, to confirm compliance with Eur. Ph. requirements, i.e., endotoxin values below 0.005 EU/ml and a pH measurement of the LAL water-reagent mixture after the test between 6 and 8.

If, as expected, the selected materials do not possess interfering activity and the results comply with Eur. Ph. specifications described above, the materials are validated. The personnel continued the validation approach, performing serial dilutions of the RSE according to the scheme described in Table 2, using LAL water and all the materials chosen for the assay.

We tested dilutions F, G, and H, which are the 3 dilutions with endotoxin content that corresponded to the 3 concentration points within the calibration curve defined by the manufacturer. Each operator used a different batch of cartridges, prepared the dilutions from the initial RSE suspension, and tested the samples in triplicate. The expected results for the analysis of dilutions F, G, and H were considered compliant if they met the criteria of Eur. Ph., the pH of the mixture was between 6 and 8, and the percentage recovery of the RSE (RSE recovery %), calculated as follows,

RSE recovery % =[Determined Value (EU/mL)/Expected Value (EU/mL)]×100

was between 50% and 200% of the expected value.

If, as expected, the result complies with specifications described above, the operators are qualified.

Finally, to be approved, the OPQ protocol should meet specific requirements for each experiment, i.e., all the correct information entered by the operator must be present in the final printed receipt and the acceptance criteria listed above must be respected.

### Matrix production

To choose potential matrices of interest to validate our LAL method, we considered the production steps of specific Epstein-Barr virus (EBV) cytotoxic T lymphocytes (CTL-EBVs), used to treat lymphoproliferative disorders.^24^^,^^25^ The method involves the initial production of the lymphoblastoid cell line (LCL) by immortalizing B lymphocytes through EBV infection, and, subsequently, CTL-EBVs are produced by co-cultivating T lymphocytes with irradiated EBV-LCL, the latter acting as antigen-presenting cells.^24^^,^^25^ An immunophenotypic characterization of >50 cell products showed that these cells were mostly CD8^+^ (68% ± 12%), CD4^+^ (19% ± 4%) and natural killer (NK) cells (7% ± 1%), while, as expected, no B cells were detected (data not shown). The ultimate goal is to treat children with PTLD. All of our in-house production processes include the first production phase, which precedes the freezing and storage of ATMPs in liquid nitrogen, and the second phase, which begins with the thawing of the cells and ends with the preparation of the final product to be infused into the patient.

As final product, we considered the cryopreserved CTL-EBVs and their preparation for the infusion into the syringe, corresponding to the formulation phase. For each cell type, we have two matrices, Matrix 1, which consists of the pre-infusion supernatant, i.e., the frozen cell washing supernatant, and Matrix 2, which represents the cell culture medium. The matrices to be analyzed should contain all the components necessary for the growth of the in-house ATMPs, ensuring that the matrices used for the validation mimic the complexity of the matrices described above (considered the worst case). All cell types are grown in complete RPMI (Lonza, Basel, Switzerland), i.e., supplemented with 10% fetal bovine serum (FBS) (HyClone, Logan, UT, USA) and 1% l-glutamine (Lonza). Moreover, LCL requires the addition of cyclosporine (Novartis, Basel, Switzerland) at a final concentration of 1.0 μg/mL, and CTLs need interleukin-2 (IL-2) at a final concentration of 400 units/mL (Novartis).^25^ All reagents and materials used for ATMP production were GMP grade. For each of the 3 independent experiments, we used a matrix derived from LCL obtained from 3 different donors, to confirm whether cells from diverse sources could affect the detection of the endotoxin in the sample.

### Validation of the analytical method and evaluation of matrix interfering activity

After completing the qualification of the instrument and operators, we proceeded with the validation of our analytical method, by evaluating the interfering activity of the matrices of our interest.

The LAL reaction is an enzymatic process,^26^ which means that, to be fully effective, it must occur within a specific range of optimal pH and with salt and bivalent cation concentration requirements. Thus, the matrices might alter these optimal conditions, rendering LAL insensitive to the endotoxins and, consequently, providing false negative results. To confirm that our matrices do not contain components that might interfere with the LAL assays, we performed an inhibition/enhancement test to determine at which dilution the samples are no longer affected by these interference factors and effects and fall within the Eur. Ph. requirements, i.e., with a PPC spike recovery of 50%–200%.^5^ Values above or under this range mean that the matrix components induce enhancement or inhibition of the test, respectively.

### Endotoxin limit and maximum validation dilution calculation for ATMP matrices

Before running the experimental protocol, it was necessary to calculate the EL and the MVD as suggested by Ph. Eur.^5^ Both EL and MVD for our in-house ATMPs were calculated as described elsewhere.^5^^,^^9^ The EL is defined as the number of EU allowed per milliliter of product, or

EL= K/M

where K is a constant equal to 5.0 EU/kg and M is the maximum dose administered per kilogram in a single-hour period that in our condition is the maximum volume of infusion per kilogram. The MVD establishes the product maximum dilution that can be performed and the EL still be detected, and it is the product of EL and concentration C divided by λ, the lowest point of the standard curve (referring to the cartridge sensitivity), i.e.,

$$\text{MVD}=\frac{\text{EL }\times \text{ C}}{\text{λ}}$$

As previously described by other authors,^9^ when assessing MVD of ATMP, concentration is equal to 1, meaning that each volume unit of the cellular suspension corresponds to one unit of product. For our in-house ATMPs, the permitted EL is not specified in the official guidelines; therefore the EL and the MVD were calculated assuming that the two matrices mentioned above represent the product to be infused.

### Matrix validation protocol

Our validation protocol involved using 3 lots of matrices, with samples used undiluted and at dilutions MVD/2 and MVD, and confirming whether the results obtained respected the Eur. Ph. acceptability criteria. If the matrix components do not induce interference because the results of the analyses at MVD/2 and MVD respect the acceptability criteria, the analyses will continue to identify the 3 non-interfering dilutions within the undiluted–MVD/2 dilution range.

Otherwise, if interferences are observed, the samples must be subjected to treatments to reduce the interferences. Once the first non-interfering dilution was identified, to ensure a valid result 2 further successive dilutions, selected within the range between undiluted and MVD/2, were analyzed. If for all 3 matrix lots the results obtained with these 3 dilution values are considered valid, the highest dilution will be chosen to be routinely tested throughout the validation.

For Matrix 1, the vial with frozen cells was thawed and the cell suspension was washed with 40 mL of 5% albumin for infusion (Grifols, Barcelona, Spain), to remove the freezing medium. The cell suspension was centrifuged and the supernatant kept to perform the quality control tests. The cell pellet was resuspended in 5% albumin for infusion, in ∼1.5–2 × 10^6^ cells/mL. For Matrix 2, cells were collected, resuspended in 45 mL of culture medium, and centrifuged. The pre-freezing supernatant was conserved for quality control tests and the cell pellet resuspended in freezing medium, i.e., PBS (Lonza), 8% FBS, and 10% DMSO (WAK-Chemie Medical GmbH, Steinbach, Germany), in ∼20–30 × 10^6^ cells/mL. The supernatants of both matrices were centrifuged for 5 min at 3,000 rpm and collected into a sterile and non-pyrogenic tube, and their pH was measured. The samples were diluted, being thoroughly homogenized for 1 min and waiting 5 min before performing each dilution and before loading it into the wells of the cartridge. Analyses were performed in duplicate for each dilution.

### pH measurement

To measure the pH at the end of the analysis, we had to consider that the total final volume of the sample recovered from the wells was small (100 μL). The operator removed the cartridge from the PTS reader, waited for the system to be ready to perform an analysis again, and re-inserted the cartridge again. This allowed the recovery and collection of the samples, which returned to the wells. Samples were pooled into a single sterile, non-pyrogenic tube, and the pH was measured with a pH indicator paper with a chromatic scale (Merck, Burlington, MA, USA), in accordance with Eur. Ph. specifications.

## Supplemental information

Document 1. Table S1 and Supplemental methods

Document 2. Article plus supplemental information
