# Complete intra-laboratory validation of a LAL assay for bacterial endotoxin determination in EBV-specific cytotoxic T lymphocytes

**DOI:** 10.1016/j.omtm.2021.11.001

**Published:** 2021-11-18

**Authors:** Salvatore Pasqua, Maria Concetta Niotta, Giuseppina Di Martino, Davide Sottile, Bruno Douradinha, Monica Miele, Francesca Timoneri, Mariangela Di Bella, Nicola Cuscino, Chiara Di Bartolo, Pier Giulio Conaldi, Danilo D’Apolito

## Main text

(Molecular Therapy: Methods & Clinical Development *22*, 320–329; September 2021)

In the originally published version of this article, Table 3 had some errors. In particular, the authors became aware of these errors when they prepared a webinar presentation in which they presented their validation work. The original Table 3 reported values for the tests performed by operator # 1, for dilutions G and H, different from the original raw data. Furthermore, by reanalyzing all the data in this table, we noticed that all the standard deviation measurements were calculated incorrectly. These have now been corrected online. The authors regret this error.

**Table 3:** Operational/Performance Qualification results

Operator	RSE dilution suspension	Spike Rxn Time CV^a^	Spike Recovery	Expected value	Measured value	Average	Standard Deviation	RSE Recovery %
QC manager	F	1.2% pass	83% pass	<0.02 EU/mL	0.016 EU/mL	0.015	1 × 10^−3^	80
		1.2% pass	88% pass	<0.02 EU/mL	0.014 EU/mL			70
		2.4% pass	59% pass	<0.02 EU/mL	0.015 EU/mL			75
	G	1.3% pass	84% pass	<0.05 EU/mL	0.039 EU/mL	0.033	5.29 × 10^−3^	78
		4.7% pass	97% pass	<0.05 EU/mL	0.029 EU/mL			58
		1.8% pass	83% pass	<0.05 EU/mL	0.031 EU/mL			62
	H	6.3% pass	97% pass	<0.01 EU/mL	0.013 EU/mL	0.013	1 × 10^−3^	130
		0.3% pass	81% pass	<0.01 EU/mL	0.014 EU/mL			140
		4.1% pass	93% pass	<0.01 EU/mL	0.012 EU/mL			120
Operator # 1	F	10.5% pass	114% pass	<0.02 EU/mL	0.018 EU/mL	0.018	5.77 × 10^−4^	90
		0.6% pass	121% pass	<0.02 EU/mL	0.019 EU/mL			95
		3.2% pass	116% pass	<0.02 EU/mL	0.018 EU/mL			90
	G	4.9% pass	90% pass	<0.05 EU/mL	0.039 EU/mL	0.044	8.96 × 10^−3^	78
		14.4% pass	142% pass	<0.05 EU/mL	0.038 EU/mL			76
		9.5% pass	72% pass	<0.05 EU/mL	0.054 EU/mL			108
	H	11.0% pass	100% pass	<0.01 EU/mL	0.009 EU/mL	0.009	0	90
		0.9% pass	98% pass	<0.01 EU/mL	0.009 EU/mL			90
		6.4% pass	88% pass	<0.01 EU/mL	0.009 EU/mL			90
Operator # 2	F	2.2% pass	104% pass	<0.02 EU/mL	0.017 EU/mL	0.017	5.77 × 10^−4^	85
		0.3% pass	92% pass	<0.02 EU/mL	0.017 EU/mL			85
		0.0% pass	93% pass	<0.02 EU/mL	0.016 EU/mL			80
	G	0.3% pass	92% pass	<0.05 EU/mL	0.055 EU/mL	0.049	6.51 × 10^−3^	110
		3.3% pass	107% pass	<0.05 EU/mL	0.042 EU/mL			84
		1.6% pass	133% pass	<0.05 EU/mL	0.049 EU/mL			98
	H	4.5% pass	83% pass	<0.01 EU/mL	0.008 EU/mL	0.010	1.53 × 10^−3^	80
		1.6% pass	105% pass	<0.01 EU/mL	0.010 EU/mL			100
		0.6% pass	158% pass	<0.01 EU/mL	0.011 EU/mL			110
Operator # 3	F	8.1% pass	71% pass	<0.02 EU/mL	0.015 EU/mL	0.021	6.03 × 10^−3^	75
		7.4% pass	78% pass	<0.02 EU/mL	0.027 EU/mL			135
		2.6% pass	113% pass	<0.02 EU/mL	0.020 EU/mL			100
	G	7.0% pass	82% pass	<0.05 EU/mL	0.046 EU/mL	0.042	4.04 × 10^−3^	92
		11.2% pass	106% pass	<0.05 EU/mL	0.043 EU/mL			86
		7.8% pass	105% pass	<0.05 EU/mL	0.038 EU/mL			76
	H	4.9% pass	60% pass	<0.01 EU/mL	0.007 EU/mL	0.010	3 × 10^−3^	70
		3.8% pass	95% pass	<0.01 EU/mL	0.013 EU/mL			130
		13.2% pass	72% pass	<0.01 EU/mL	0.010 EU/mL			100

a Spike Rxn Time CV, spike reaction time coefficient of variation

