# Supplementary material for: Complete intra-laboratory validation of a LAL assay for bacterial endotoxin determination in EBV-specific cytotoxic T lymphocytes
Source: Mol Ther Methods Clin Dev. 2021 May 14;22:320–9. doi: 10.1016/j.omtm.2021.05.002 (PMC8408548; doi:10.1016/j.omtm.2021.05.002)
Supplement: Document 1. Table S1 and Supplemental methods [file mmc1.pdf]

OMTM, Volume *22*

Supplemental information

Complete intra-laboratory validation of a LAL assay for bacterial endotoxin determination in EBV-specific cytotoxic T lymphocytes

Salvatore Pasqua, Maria Concetta Niotta, Giuseppina Di Martino, Davide Sottile, Bruno Douradinha, Monica Miele, Francesca Timoneri, Mariangela Di Bella, Nicola Cuscino, Chiara Di Bartolo, Pier Giulio Conaldi, and Danilo D'Apolito

Supplemental table

Table S1. Risk Priority Numbers (RPNs) of failure modes for validation protocol and respective highest RPN and RPN after corrective action

Step	Failure mode	Possible effect	Possible cause	Possible mode of detection	Estimated frequency of occurrence (O)	Estimated frequency of detection (D)	Estimated severity (S)	RPN	Corrective action	RPN after corrective action	Improvement index[a]
1	Instrument is not present	We cannot perform the validation process	The supplier has not sent the instrument yet/Delay in the expedition	Visual inspection	1	1	8	8	Call the company to have the instrument replaced	8	1
	Certified documents are not present	We cannot perform the validation process	The supplier forgot to send them	Visual inspection	1	1	6	6	Call the company and ask them to send all the documentation	6	1
	Instrument does not power up	We cannot perform the validation process	Broken during expedition	Power-up test of the instrument	1	1	8	8	Call the company to have the instrument replaced	8	1
2	The operator does not qualifies for training verification	Delay in validation time schedule	Inadequate training	Test verification	1	1	7	7	Repeat the training	7	1
3	Instrument malfunction	The software does not work	Defective software	Visual inspection	1	1	8	8	Call the company to replace the equipment and/or software	8	1
	Bubble formation inside the wells	The test is not valid	The operators do not dispense the sample in the correct manner	Visual inspection of the wells	1	5	6	30	Repeat the training	5	6
	The expected results for the LAL water are out of the acceptance criteria	The test is not valid	The selected materials display interferences	Reading the effective endotoxin value in the receipt	1	1	8	8	Change the selected materials and repeat the tests	8	1
	The correct information entered by the operator was not present on the receipt	The instrument is not suitable for GMP applications	The software does not work as described	Reading the information in the receipt	1	1	9	9	Call the company to replace the equipment and/or software	9	1
	The expected results for the analysis of dilutions F, G and H are out of the acceptance criteria	The test is not valid	Serial dilutions of the Reference Standard Endotoxin (RSE) were prepared incorrectly	Reading effective endotoxin values in the receipt	1	1	8	8	Repeat the training and the Qualification of Operators	8	1
4	The results of Matrix 1 analysis do not respect the acceptability criteria at MVD/2 and MVD	It is not possible to perform the validation	The matrix shows interference	Spike recovery is out of the acceptance range	10 (after corrective action 1)	1	10	100	Use of suitable treatment to remove interferences and repeat the matrix validation protocol	10	10
5	The results of Matrix 2 analysis do not respect the acceptability criteria at MVD/2 and MVD	It is not possible to perform the validation	The matrix shows interference	Spike recovery is out of the acceptance range	1	1	10	10	Use of suitable treatment to remove interferences and repeat the matrix validation protocol	10	1

[a] RPN/RPN after corrective action

Supplemental Methods

Risk analysis

The measurement of endotoxins is a key analytical parameter to quantify impurities in an ATMP product. To mitigate the risk of adverse events, regulatory agencies request producers to guarantee low levels of endotoxins. To assure product quality and consistency, the chapter 2.6.14 of Ph. Eur. [1] describes all the methods accepted to measure the endotoxinic content in pharmaceutical products. It is important to carefully select the analytical test to quantify the amount of endotoxins in these products.

As suggested by regulatory agencies, to ensure reliability of our analytical method and its validation, we identified all possible risks to the consistent and robust performance of the entire process. To evaluate the quality of the validation protocol, we took into account several of its points, evaluating the intrinsic risks to the methodology. [2] As performed by others, we used the Failure Mode and Effects Analysis (FMEA). [3–5]

Our team divided the analytical method validation into the following individual steps:

1. Installation qualification
2. Training
3. Validation of material, Operational/Performance Qualification and Qualification of Operators
4. Matrix 1 study
5. Matrix 2 study

Subsequently, failure modes were identified for each step. For each failure mode, the frequency of occurrence (O), the probability that the failure would remain undetected (D) and its severity (S) were estimated, giving each of these parameters a value from 1 to 10, where the highest the number, higher the risk. The value associated for each parameter was determined by a consensus decision of the team. For each identified failure mode, we calculated the Risk Priority Number (RPN) by the following formula:

$$\mathrm{RPN} = \mathrm{O} \times \mathrm{D} \times \mathrm{S}$$

Table S1 summarizes our risk analysis, which strengthens that the use of PTS™ is a good choice to obtain quality control results which respect the requirements stipulated by the GMP guidelines. Also, this instrument ensures a very low level of failure mode regarding its performance and personnel, and the risk analysis allowed the improvement of the validation method by identifying, evaluating and correcting specific failure modes.

References

1. European Pharmacopoeia (2017). 2.6.14 Bacterial Endotoxins. In European Pharmacopoeia, pp. 171–175.
2. Agency, E.M. (2013). Guideline on the risk-based approach according to annex I, part IV of Directive 2001/83/EC applied to Advanced therapy medicinal products.
3. van Leeuwen, J.F., Nauta, M.J., de Kaste, D., Odekerken-Rombouts, Y.M.C.F., Oldenhof, M.T., Vredenbregt, M.J., and Barends, D.M. (2009). Risk analysis by FMEA as an element of analytical validation. J Pharm Biomed Anal *50*, 1085–1087.
4. Dailey, K.W. (2004). The FMEA Pocket Handbook M. Minturn, D. Wieckhorst, and B. Welch, eds. (, DW Publishing Co.).
5. Agency, E.M. (2015). ICH guideline Q9 on quality risk management.
